# Supplementary material for: Modeling glioblastoma heterogeneity as a dynamic network of cell states
Source: Mol Syst Biol. 2021 Sep 16;17(9):e10105. doi: 10.15252/msb.202010105 (PMC8444284; doi:10.15252/msb.202010105)
Supplement: Supplementary file 6 — Source Data for Figure 5 [file MSB-17-e10105-s004.zip › Figure5A_sourcedata/GSEA_3017/nosigdb_stateB.GseaPreranked.1622795021725/NB_KI_CLUSTER5.html]

Details for gene set NB\_KI\_CLUSTER5[GSEA]

|  || Dataset | state43017 |
| Phenotype | NoPhenotypeAvailable |
| Upregulated in class | na\_pos |
| GeneSet | NB\_KI\_CLUSTER5 |
| Enrichment Score (ES) | 0.57696533 |
| Normalized Enrichment Score (NES) | 3.0953794 |
| Nominal p-value | 0.0 |
| FDR q-value | 0.0 |
| FWER p-Value | 0.0 |
Table: GSEA Results Summary

  

Fig 1: Enrichment plot: NB\_KI\_CLUSTER5      
 Profile of the Running ES Score & Positions of GeneSet Members on the Rank Ordered List

  

| PROBE | GENE SYMBOL | GENE\_TITLE | RANK IN GENE LIST | RANK METRIC SCORE | RUNNING ES | CORE ENRICHMENT || 1 | HIST1H4C |  |  | 0 | 1.373 | 0.0397 | Yes |
| 2 | HIST1H1C |  |  | 4 | 1.041 | 0.0655 | Yes |
| 3 | HIST1H1E |  |  | 5 | 0.983 | 0.0939 | Yes |
| 4 | HIST2H2AC |  |  | 9 | 0.872 | 0.1148 | Yes |
| 5 | HMGB2 |  |  | 14 | 0.786 | 0.1318 | Yes |
| 6 | FBXO5 |  |  | 15 | 0.785 | 0.1545 | Yes |
| 7 | PRC1 |  |  | 18 | 0.756 | 0.1735 | Yes |
| 8 | ATAD2 |  |  | 28 | 0.685 | 0.1803 | Yes |
| 9 | PCLAF |  |  | 31 | 0.681 | 0.1971 | Yes |
| 10 | KPNA2 |  |  | 33 | 0.676 | 0.2152 | Yes |
| 11 | SGO2 |  |  | 36 | 0.664 | 0.2316 | Yes |
| 12 | SMC4 |  |  | 41 | 0.655 | 0.2447 | Yes |
| 13 | PRR11 |  |  | 45 | 0.649 | 0.2592 | Yes |
| 14 | DHFR |  |  | 49 | 0.638 | 0.2733 | Yes |
| 15 | CKAP2 |  |  | 53 | 0.628 | 0.2871 | Yes |
| 16 | CENPK |  |  | 62 | 0.603 | 0.2930 | Yes |
| 17 | SMC2 |  |  | 68 | 0.586 | 0.3028 | Yes |
| 18 | CDK1 |  |  | 69 | 0.585 | 0.3197 | Yes |
| 19 | CKS1B |  |  | 72 | 0.575 | 0.3334 | Yes |
| 20 | CIP2A |  |  | 73 | 0.574 | 0.3500 | Yes |
| 21 | RACGAP1 |  |  | 75 | 0.572 | 0.3651 | Yes |
| 22 | PLK1 |  |  | 78 | 0.567 | 0.3786 | Yes |
| 23 | DBF4 |  |  | 79 | 0.567 | 0.3950 | Yes |
| 24 | CKS2 |  |  | 83 | 0.558 | 0.4068 | Yes |
| 25 | ECT2 |  |  | 84 | 0.558 | 0.4230 | Yes |
| 26 | UBE2S |  |  | 87 | 0.555 | 0.4362 | Yes |
| 27 | CENPM |  |  | 92 | 0.544 | 0.4461 | Yes |
| 28 | MXD3 |  |  | 104 | 0.519 | 0.4453 | Yes |
| 29 | UBE2T |  |  | 110 | 0.512 | 0.4528 | Yes |
| 30 | DIAPH3 |  |  | 112 | 0.510 | 0.4661 | Yes |
| 31 | TUBB4B |  |  | 114 | 0.507 | 0.4793 | Yes |
| 32 | BIRC5 |  |  | 116 | 0.504 | 0.4925 | Yes |
| 33 | CLSPN |  |  | 117 | 0.503 | 0.5070 | Yes |
| 34 | CEP152 |  |  | 119 | 0.502 | 0.5201 | Yes |
| 35 | DUT |  |  | 124 | 0.494 | 0.5286 | Yes |
| 36 | SPDL1 |  |  | 126 | 0.492 | 0.5414 | Yes |
| 37 | SHCBP1 |  |  | 133 | 0.484 | 0.5468 | Yes |
| 38 | GMNN |  |  | 135 | 0.484 | 0.5593 | Yes |
| 39 | NCAPD2 |  |  | 136 | 0.483 | 0.5733 | Yes |
| 40 | BRCA1 |  |  | 171 | 0.440 | 0.5369 | Yes |
| 41 | MIS18BP1 |  |  | 186 | 0.430 | 0.5292 | Yes |
| 42 | CDKN2D |  |  | 190 | 0.427 | 0.5372 | Yes |
| 43 | TCF19 |  |  | 207 | 0.410 | 0.5259 | Yes |
| 44 | ZWINT |  |  | 212 | 0.407 | 0.5319 | Yes |
| 45 | LIG1 |  |  | 214 | 0.407 | 0.5423 | Yes |
| 46 | H2AFZ |  |  | 216 | 0.405 | 0.5525 | Yes |
| 47 | FANCA |  |  | 227 | 0.395 | 0.5495 | Yes |
| 48 | EZH2 |  |  | 236 | 0.388 | 0.5492 | Yes |
| 49 | TRIP13 |  |  | 241 | 0.387 | 0.5546 | Yes |
| 50 | PCNA |  |  | 242 | 0.386 | 0.5658 | Yes |
| 51 | HMGN2 |  |  | 243 | 0.386 | 0.5770 | Yes |
| 52 | CENPW |  |  | 267 | 0.366 | 0.5543 | No |
| 53 | CHAF1A |  |  | 276 | 0.359 | 0.5532 | No |
| 54 | ANP32E |  |  | 280 | 0.358 | 0.5592 | No |
| 55 | CDC25B |  |  | 286 | 0.356 | 0.5623 | No |
| 56 | ZWILCH |  |  | 292 | 0.353 | 0.5653 | No |
| 57 | DEK |  |  | 316 | 0.341 | 0.5420 | No |
| 58 | RHEB |  |  | 404 | 0.307 | 0.4253 | No |
| 59 | PTTG1 |  |  | 413 | 0.302 | 0.4225 | No |
| 60 | TEX30 |  |  | 448 | 0.294 | 0.3819 | No |
| 61 | MZT1 |  |  | 456 | 0.291 | 0.3802 | No |
| 62 | CHEK1 |  |  | 477 | 0.286 | 0.3596 | No |
| 63 | NUCKS1 |  |  | 483 | 0.285 | 0.3607 | No |
| 64 | CDT1 |  |  | 486 | 0.284 | 0.3660 | No |
| 65 | DNMT1 |  |  | 523 | 0.277 | 0.3221 | No |
| 66 | GGH |  |  | 564 | 0.270 | 0.2721 | No |
| 67 | DDX11 |  |  | 568 | 0.269 | 0.2756 | No |
Table: GSEA details [plain text format]

  

Fig 2: NB\_KI\_CLUSTER5: Random ES distribution      
 Gene set null distribution of ES for **NB\_KI\_CLUSTER5**
